# Prevalence and associated risk factors for dry eye disease among Brazilian undergraduate students

**DOI:** 10.1371/journal.pone.0259399

**Published:** 2021-11-11

**Authors:** Isabela Yang, Tais Wakamatsu, Isabella Batistela Inhesta Sacho, José Henrique Fazzi, Asafe César de Aquino, Gabriel Ayub, Pedro Albuquerque Rebello, José Álvaro Pereira Gomes, Monica Alves

**Affiliations:** 1 Department of Ophthalmology and Otorhinolaryngology, School of Medical Sciences, University of Campinas (UNICAMP), Campinas, São Paulo, Brazil; 2 Department of Ophthalmology and Visual Sciences, Paulista School of Medicine, Federal University of São Paulo (UNIFESP), São Paulo, São Paulo, Brazil; Saarland University, GERMANY

## Abstract

**Purpose:**

Dry eye is a common, complex, and multifactorial disease of the ocular surface and tear film that results in discomfort and visual disturbances. Prevalence rates vary and largely rely on studies involving older populations. This study sought to evaluate dry eye among a sample of young students in Brazil.

**Methods:**

Cross-sectional survey included 2,140 students using 2 self-applicable questionnaires of dry eye symptoms: the Ocular Surface Disease Index (OSDI) and the Women’s Health Study (WHS) questionnaire and a list of risk factors associated with dry eye. Participants with dry eye symptoms underwent a clinical evaluation.

**Results:**

Participants were 23.4±5.2 years of age, 56.1% female and 43.9% male, 34.4% had an OSDI score greater than 22, and 23.5% had dry eye according to the WHS. Dry eye frequency differed consistently between the sexes: 42.6% women and 24.0% men based on the OSDI, and 27.1% women and 18.5% men based on the WHS. Univariate and multivariate analyses demonstrated that female sex, contact lens wear, the screen use for more than 6 hours per day, less than 6 hours of sleep a night, and certain medications were relevant related risk factors for dry eye. Despite symptoms, clinical evaluations demonstrated mild signs of dry eye.

**Conclusions:**

Dry eye symptoms were found to be a prevalent condition among Brazilian undergraduate students. Compared to the rates of dry eye among the general Brazilian population over 40 years of age, students present at higher dry eye symptoms rates and distinct odds for related risk factors were identified.

## Introduction

Dry eye disease (DED) is a multifactorial disease of the ocular surface commonly presented in clinical practice. Symptom quantification combined with an evaluation of clinical signs is crucial for the achievement of a proper diagnosis and for creating a true picture of disease magnitude [1–4]. The Tear Film and Ocular Surface Society (TFOS) Society Dry Eye Workshop (DEWS) II defines DED as "a multifactorial disease of the ocular surface characterized by a loss of homeostasis of the tear film and accompanied by ocular symptoms, in which tear film instability and hyperosmolarity, ocular surface inflammation and damage neurosensory abnormalities play etiological roles” [2]. This broad definition highlights the most relevant aspects of this disease, the complexity of its mechanisms, and its possible impact on both the ocular surface and patients’ quality of life.

A variety of questionnaires and clinical evaluations have been applied to understand the epidemiology of DED, but there is significant variation between studies [4, 5]. The overall prevalence of DED ranges from 5% to 50% depending on the criteria, age, sex, and population studied [4]. In women, rates of DED have been found to be 1.33 to 1.74 times higher than in men; it is usually more common in Asian populations than in Caucasian populations, and prevalence rates increase with age. The TFOS DEWS II epidemiology committee has provided a meta-analysis to determine the prevalence of dry eye based on different diagnostic criteria and stratified by age and sex [4]. The prevalence of symptomatic and clinically diagnosed dry eye was found to vary by age and sex, but only one study included young participants [4, 6]. Recently, some reports have evaluated DED in younger populations [5, 7, 8]. A Japanese study evaluated 3,433 high school students between 15 and 18 years of age and found a prevalence of clinically diagnosed DED of 4.3% in boys and 8.0% in girls [6]. In China, the prevalence of DED in high school students has been found to be 23.7% [9]. Distinct risk factors may contribute to DED in this age group, including longtime use of electronic devices, contact lens wear, medications (such as oral contraceptives, antidepressants, or isotretinoin derivates), and sleep deprivation [5, 10]. Epidemiological studies on multifactorial diseases such as DED are necessary to recognize underlying risk factors and shed light on possible prevention strategies and treatments.

In Brazil, a previous study using the Women’s Health Study (WHS) Dry Eye Symptom Questionnaire reported an overall DED prevalence of 12.8% in a population over 40 years of age [11]. The country is diverse in terms of climate and socioeconomics. If the findings on previous study’s cohort of 3,107 participants from the country’s five geopolitical regions can be extrapolated to Brazil as a whole (more than 210 million inhabitants), almost 27 million are likely to have DED symptoms. It is important to note, however, that there is no information on prevalence among younger age groups. The current study sought to assess prevalence, related risk factors, and clinical signs of DED in a young population in Brazil.

## Methods

### Study population

The cross-sectional two-center study evaluated DED symptoms through the application of translated and validated versions of the OSDI [12] and WHS questionnaires [13] to undergraduate students enrolled at either the University of Campinas (UNICAMP) or the Federal University of São Paulo (UNIFESP) in Brazil. A list of risk factors was included in the survey. Participants who reported DED symptoms were invited to undergo a detailed clinical evaluation at the Ophthalmology Department of each institution. The study was approved by the ethics committees of both universities and was conducted in accordance with the Declaration of Helsinki. Informed consent was obtained from all participants and parents or guardians of the minors.

The questionnaires were distributed in two different ways: a printed version was distributed during regular in-person classes, and an electronic form that was sent via an institutional e-mail. Students were assured that participation in the research would not interfere with academic activities. The informed consent form and a list of potential risk factors were included with the questionnaires. All instructions were provided either in person or electronically, depending on the type of questionnaire completed.

### Dry eye symptom questionnaire assessment

The WHS Dry Eye Questionnaire consists of three simple and direct questions. Volunteers respond to the frequency with which their eyes feel dry and the frequency with which their eyes feel irritated. They then report whether they have received a previous diagnosis of dry eye. Volunteers then select which risk factors apply to them.

The risk factors listed include contact lens wear, previous ocular surgery, the use of devices with screens (computer, tablets and/or mobile phone) for more than 6 hours per day, medications such as oral contraceptives, antidepressants, anti-allergy medications, and isotretinoin, limited sleep, smoking, current or prior cancer treatment, and comorbidities such as diabetes and rheumatological disease (including rheumatoid arthritis, systemic lupus erythematosus, and collagen disease).

The OSDI score is a subjective symptom questionnaire that includes 12 items on visual function, symptoms, and environment; volunteers grade each symptom on a 0-to-4 scale. The score algorithm ranges from 0 (no disability) to 100 (complete disability); a score greater than or equal to 22 points is considered representative of moderate or severe DED [4].

### Clinical evaluation

All participants who scored greater than or equal to 22 on the OSDI and/or who reported severe symptoms of dry eye or previous diagnosis as per the WHS questionnaire were invited to receive a clinical evaluation. The clinical exam encompassed a detailed evaluation of the ocular surface to assess meibomian gland expression and tear break-up time, as well as fluorescein and green lissamine staining and Schirmer’s test.

Non-invasive tear film break-up time (NITBUT) was evaluated using the Keratograph 5M (Oculus, Wetzlar, Germany) software, which provides an automated assessment of tear stability. It measures the interval of time between three naturally complete blinks and the appearance of the first break in the tear film after ceased blinking. A cut-off value of less than or equal to 10 s has been established. NITBUT has become more popular as an assessment tool in recent years since tear film stability can be affected by fluorescein.

Meibomian gland expressibility was graded based on the number of expressible glands, which itself was determined by the application of digital pressure to the four glands on the central portion of the inferior eyelid. Our grading scheme was as follows: grade 0 referred to all glands; grade 1 referred to 3 to 4 glands; grade 2 referred to 1 to 2 glands, and grade 3 referred to cases in which no glands were expressible. Meibum quality was also determined according to a grading scheme, in which grade 0 referred to translucent oil secretion, grade 1 referred to opaque secretion, grade 2 referred to opaque secretion with granules, and grade 3 referred to pasty secretion.

Fluorescein staining of the ocular surface was graded according to the National Eye Institute (EU) Industry Workshop guidelines [14]. This scale divides the cornea into five areas and grades the staining from 0 (absent) to 3 (severe) in each of them, with a total score varying from 0 to 15. Lissamine green stains damage epithelial cells. It was evaluated according to the Oxford grading scale, on which scores range from 0 to 9, same as described above (0–3) but considering 3 areas of the ocular surface (nasal, central and temporal) [15].

Schirmer’s test without anesthesia was performed with standardized strips of filter paper that were placed in the lateral canthus away from the cornea and left in place for five minutes with the eyes closed. Readings were recorded in millimeters of wetting for five minutes. A reading of less than 10 mm was referred as an aqueous deficiency.

### Statistical analysis

Data was analyzed using the Statistical Analysis System (SAS) for Windows, version 9.4. (SAS Institute Inc., Cary, NC, USA) and GraphPad Prism, version 9.0.0 (86) (California, CA, USA). Exploratory data analysis was performed through summary measures (mean, standard deviation and frequency). Intergroup comparisons of continuous measurements were performed using the Mann-Whitney U test, and categorical data was analyzed using the chi-square test or Fisher’s exact test. To identify risk factors associated with DED, the odds ratio (OR) and 95% confidence intervals (CI) were calculated using the univariate and multivariate logistic regression models. All analyses were separated by sex. A P-value less than 0.05 was considered statistically significant.

## Results

### Study population

A total of 485 UNICAMP undergraduate students and 491 UNIFESP medical student volunteers completed the questionnaires in class, and another 1,182 students completed the online version, producing a total of 2,158 participants. Participants who submitted incomplete questionnaires were excluded, and 2,140 participants remained. Of these, 1,200 (56.07%) were female and 940 (43.93%) were male, with a total median age of 22 years (range 16–55 years) and a mean age of 23.44 ± 5.2 years. Of the volunteers included, 1,649 were undergraduate students from UNICAMP enrolled in different degree programs, and the 491 volunteers from UNIFESP were medical students. When the students were grouped by degree program, 1128 students (52.7%) were enrolled in programs in the biological sciences, 699 students (32.7%) were enrolled in programs in the exact sciences, and 313 students (14.6%) were enrolled in humanities programs.

### Dry eye disease prevalence

**Fig 1:**
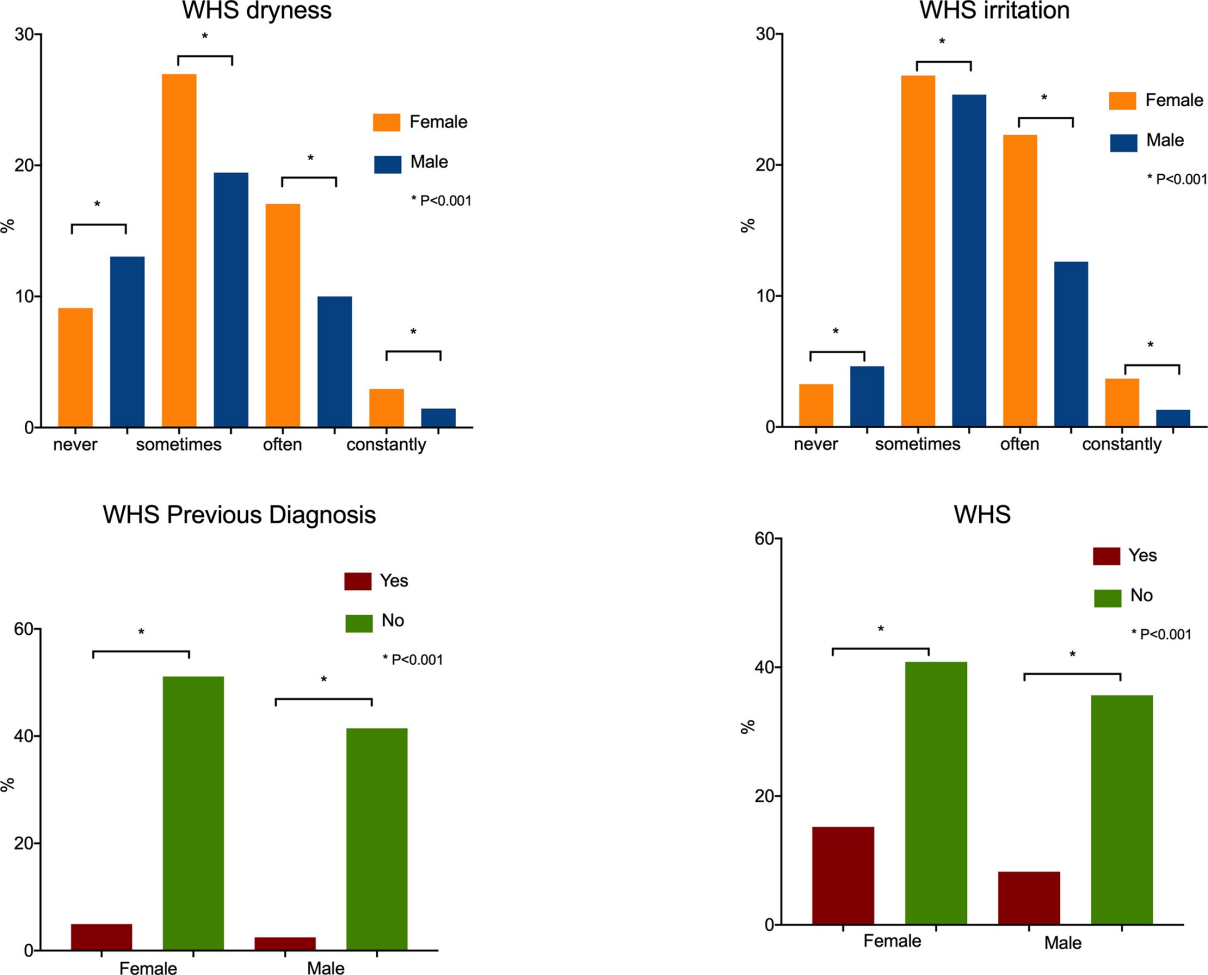
Sex distribution of dry eye symptom frequency and previous DED diagnosis among university students according to responses to the dry eye symptom questionnaire used in the Women’s Health Study (WHS). Data expressed as a frequency (%). WHS: Women Heath Study.

**Fig 2:**
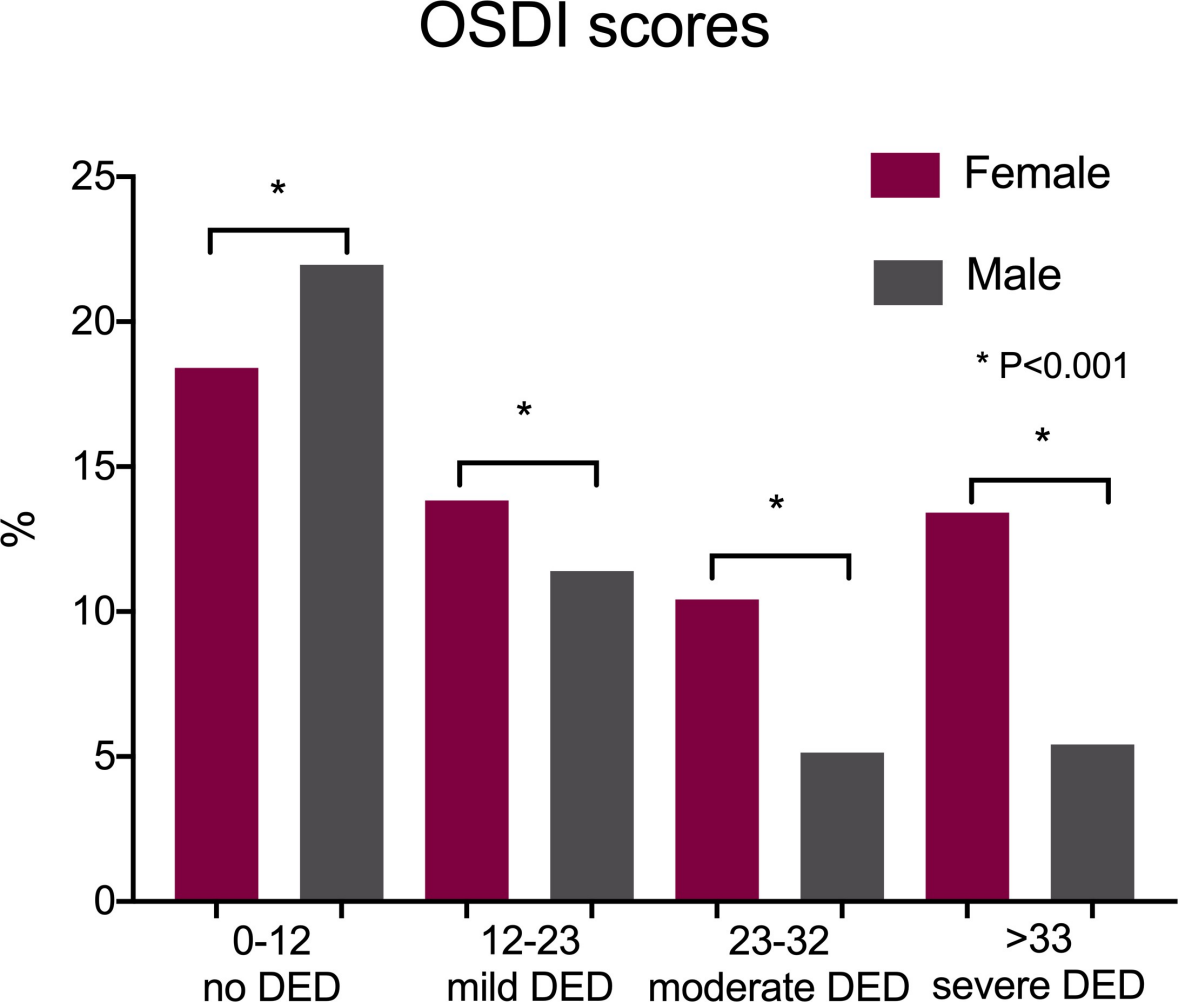
Title distribution of Ocular Surface Disease Index (OSDI) scores by sex. Data expressed as a frequency (%). OSDI: Ocular Surface Disease Index.

According to the OSDI results, 59.64% of the volunteers (1,277/2,140) scored greater than 12 (representative of some level of DED); 34.39% of the volunteers (736/2,140) scored greater than or equal to 22 (a score representing moderate or severe DED). According to the WHS questionnaire, 23.5% (503/2,140) had severe dry eye and/or a DED diagnosis. The distribution of responses is displayed in Table 1. Figs 1 and 2 demonstrate dry eye distribution by sex for both the WHS and OSDI questionnaire.

**Table 1:** Distribution of responses and frequencies of DED according to the WHS and OSDI questionnaires.

	WHS		OSDI
**Irritation**	Never: 22.1% (474/2,140)	**No DED (score 0–12)**	40.37% (864/2,140)
	Sometimes: 46.4% (993/2,140)		
	Often: 27.1% (579/2,140)		
	Constantly: 4.4% (94) /2,140		
**Dryness**	Never: 7.9% (169/2,140)	**Mild (score 13–22)**	25.23% (540/2,140)
	Sometimes: 52.2% (1,117/2,140)		
	Often: 34.9% (747/2,140)		
	Constantly: 5% (105) /2,140		
**Previous diagnosis**	7.43% (159/2,140)	**Moderate (score 23–32)**	15.56% (333/2,140)
		**Severe (score >33)**	18.83% (403/2,140)
**Total DED**	23.5% (503/2,140)	**Total DED**	34.39% (736/2,140)
**Severe symptoms and/or previous diagnosis**	Female 64.8% (326/503)	OSDI score > 22	Female 69.29% (510/736)
	Male 35.2% (177/503)		Male 30.71% (226/736)

Data expressed as frequency (%). WHS: Women’s Health Questionnaire; OSDI: Ocular Surface Disease Index; DED: Dry Eye Disease.

### Dry eye disease risk factors

Table 2 displays demographic data and risk factors, and Table 3 details the risk factor distribution according to each questionnaire.

**Table 2:** Demographics and frequency of DED risk factors of the study population (n = 2,140).

Sex (female/male)	1200/940
Site (Campinas/São Paulo)	1,649/491
Age in years (mean ±SD)	23.44±5.2
More than 6 hours per day of screen time	69.3% (1,483)
Less than 6 hours of sleep per night	38% (792)
Oral contraceptive use	39% (471/1200)
Contact lens wear	16.4% (351)
Anti-depressant use	15% (321)
Anti-allergy medication use	10.4% (222)
Smoking	5.6% (120)
Isotretinoin use	2.6% (56)
History of ocular surgery	2.6% (56)
Diabetes	0.8% (17)
Rheumatological disease	0.7% (16)
Cancer treatment	0.3% (6)

Data expressed as frequency (%). SD: Standard deviation.

**Table 3:** Risk factors of dry eye disease (DED) according to the Dry Eye Syndrome Questionnaire used in the Women’s Health Study (WHS) and the Ocular Surface Disease Index (OSDI).

Variable		WHS DED	No WHS DED	P-Value	OSDI>22	OSDI<22	P-value
Sex	Female	64.8%	53.4%	**<0.01** ^**1**^	69.3%	49.2%	**<0.01** ^**1**^
	Male	35.2%	46.6%		30.7%	50.8%	
Contact lens wear	Yes	21.7%	14.8%	**<0.01** ^**1**^	17.7%	15.8%	0.26^**1**^
	No	78.3%	85.2%		82.3%	84.2%	
Ocular surgery	Yes	4.8%	2.0%	**<0.01** ^**1**^	3.5%	2.1%	**0.05** ^**1**^
	No	95.2%	98.0%		96.5%	97.9%	
Screen time> 6 h/day	Yes	81.1%	65.7%	**<0.01** ^**1**^	81.7%	62.9%	**<0.01** ^**1**^
	No	18.9%	34.3%		18.3%	37.1%	
Oral contraceptive	Yes	24.9%	21.1%	0.08^**1**^	26.0%	20.0%	**<0.01** ^**1**^
	No	75.1%	78.9%		74.0%	80.0%	
Antidepressant	Yes	10.9%	10.3%	0.69^**1**^	12.8%	9.2%	**0.01** ^**1**^
	No	89.1%	89.7%		87.2%	90.8%	
Anti-allergy	Yes	20.7%	13.4%	**<0.01** ^**1**^	20.1%	12.6%	**<0.01** ^**1**^
	No	79.3%	86.6%		79.9%	87.4%	
Isotretinoin	Yes	4.6%	2.1%	**<0.01** ^**1**^	3.3%	2.4%	0.21^**1**^
	No	95.4%	97.9%		96.7%	97.6%	
Diabetes	Yes	0.8%	0.9%	0.89^**1**^	1.2%	0.6%	0.16^**1**^
	No	99.2%	99.1%		98.8%	99.4%	
Rheumatological disease	Yes	1.6%	0.5%	**0.03** ^**1**^	1.8%	0.3%	**<0.01** ^**1**^
	No	98.4%	99.5%		98.2%	99.7%	
Cancer treatment	Yes	0.4%	0.3%	0.67^2^	0.5%	0.2%	0.24^2^
	No	99.6%	99.7%		99.5%	99.8%	
Smoking	Yes	7.2%	5.2%	0.09^**1**^	7.6%	4.6%	**<0.01** ^**1**^
	No	92.8%	94.8%		92.4%	95.4%	
Hours of sleep	3–6 hours	40.8%	37.1%	0.14^**1**^	42.2%	35.7%	**<0.01** ^**1**^
	>6 hours	59.2%	62.9%		57.8%	64.3%	

^1^Based on the Chi-Square test

^2^Based on Fisher’s exact test. OSDI: Ocular Surface Disease Index; WHS: Women’s Health Study; DED: Dry eye disease.

Univariate and multivariate analyses were performed using logistic regression models and are displayed in Table 4. Based on the results from the two questionnaires, the main independent risk factors associated with DED in the univariate and multivariate logistic regression analyses in this young Brazilian study population were female sex, screen use, anti-allergy medication use, and rheumatological disease. Meanwhile, certain variables, such as ocular surgery, contact lens wear, and isotretinoin use, were associated with DED according only to the WHS results, and contraceptive and antidepressant use, smoking, and less than 6 hours of sleep per day were associated with DED according only to the OSDI. Tables 5 and 6 display the subgroups of females and males, respectively, and detail the independent risk factors associated with DED according to the multivariate logistic regression analysis. It is important to note that, when females were considered separately, the independent factors associated with DED remained the same. In the male subgroup, only screen use for more than 6 hours per day was found to be associated with both sets of DED criteria; antidepressant and anti-allergy medication use were associated with DED only in the WHS results, and ocular surgery and less than 6 hours per day of sleep were associated with DED based only on the OSDI results.

**Table 4:** Associations of DED risk factors using logistic regression models in univariate analyses and multivariate analyses.

	Univariate Analysis						Multivariate Analysis					
Risk Factor	DED as per WHS			OSDI > 22			DED as per WHS			OSDI > 22		
	OR	95% CI	P-Value	OR	95% CI	P-Value	*OR*	95% CI	P-Value	OR	95% CI	P-Value
Female	1.61	1.31–1.98	**<0.01**	2.33	1.93–2.82	**<0.01**	*1*.*51*	1.17–1.91	**<0.01**	2.31	1.86–2.86	**<0.01**
Contact lens wear	1.59	1.240–2.04	**<0.01**	1.15	0.90–1.46	0.2	*1*.*73*	1.31–2.25	**<0.01**	1.24	0.96–1.60	0.10
Ocular Surgery	2.50	1.46–4.30	**<0.01**	1.68	0.97–2.8	0.06	*2*.*72*	1.53–4.76	**<0.01**	1.83	1.04–3.20	0.04
Screen > 6 h/day	2.24	1.76–2.87	**<0.01**	2.63	2.11–3.25	**<0.01**	*2*.*28*	1.76–2.956	**<0.01**	2.40	1.95–3.12	**<0.01**
Oral contraceptive	1.22	0.98–1.56	0.08	1.41	1.14–1.74	**<0.01**	*0*.*87*	0.67–1.15	0.34	0.77	0.61–1.00	0.05
Antidepressant	1.07	0.76–1.46	0.69	1.43	1.07–1.90	**<0.01**	*0*.*93*	0.676–1.32	0.72	1.15	0.84–1.55	0.34
Anti-allergy	1.68	1.29–2.17	**<0.01**	1.76	1.37–2.22	**<0.01**	*1*.*52*	1.156–1.98	**<0.01**	1.45	1.12–1.89	**<0.01**
Isotretinoin	2.26	1.32–3.86	**<0.01**	1.40	0.81–2.39	0.21	*2*.*23*	1.25–3.98	**<0.01**	1.64	0.92–2.92	0.09
Diabetes	0.92	0.30–2.84	0.89	1.92	0.76–4.85	0.17	*0*.*96*	0.29–3.06	0.94	2.17	0.83–5.72	0.10
Rheumatological disease	2.91	1.11–7.61	**0.03**	6.29	2.03–19.36	**<0.01**	*3*.*15*	1.13–8.66	**<0.01**	4.95	1.54–15.99	**<0.01**
Cancer treatment	1.30	0.24–6.73	0.74	2.55	0.57–11.43	0.21	*1*.*42*	0.25–7.90	0.68	3.12	0.64–15.27	0.15
Smoking	1.41	0.943–2.11	0.09	1.69	1.16–2.44	**<0.01**	*1*.*26*	0.82–1.93	0.26	1.43	0.97–2.13	0.06
< 6 hours of sleep	1.17	0.94–1.42	0.14	1.31	1.08–1.57	**<0.01**	*1*.*13*	0.91–1.40	0.22	1.27	1.04–1.55	**<0.01**

OR: Odds Ratio, CI: Confidence Interval, OSDI: Ocular Surface Disease Index; WHS: Women’s Health Study; DED: Dry eye disease.

**Table 5:** Associations of DED risk factors in female subgroup, using logistic regression models in multivariate analyses.

Risk Factor	DED as per WHS			OSDI > 22		
	OR	95% CI	P-Value	OR	95% CI	P-Value
Contact lens wear	1.87	1.36–2.61	**<0.01**	1.28	0.94–1.74	0.12
Ocular Surgery	3.66	1.81–7.39	**<0.01**	1.22	0.60–2.49	0.56
Screen time > 6 h/day	2.05	1.45–2.85	**<0.01**	2.30	1.71–3.10	**<0.01**
Oral contraceptive	0.87	0.67–1.16	0.37	0.77	0.60–0.99	**0.04**
Antidepressant	0.88	0.58–1.30	0.52	1.16	0.813–1.64	0.40
Anti-allergy	1.40	1.02–1.96	**<0.01**	1.50	1.12–2.05	**<0.01**
Isotretinoin	1.33	0.53–3.40	0.54	1.69	0.68–4.11	0.24
Diabetes	1.55	0.39–6.28	0.53	1.72	0.47–6.21	0.41
Rheumatological disease	4.12	1.37–12.25	**<0.01**	5.51	1.49–20.66	**<0.01**
Cancer treatment	0.91	0.09–9.19	0.9346	4.57	0.45–46.71	0.20
Smoking	1.34	0.75–2.45	0.31	1.29	0.73–2.28	0.36
< 6 hours of sleep	1.01	0.78–1.34	0.87	1.22	0.95–1.56	0.09

OR: Odds Ratio, CI: Confidence Interval, OSDI: Ocular Surface Disease Index; WHS: Women’s Health Study; DED: Dry eye disease.

**Table 6:** Associations of DED risk factors in male subgroup, using logistic regression models in multivariate analyses.

Risk Factor	DED as per WHS			OSDI > 22		
	OR	95% CI	P-Value	OR	95% CI	P-Value
Contact lens	1.46	0.89–2.38	0.12	1.11	0.68–1.77	0.67
Ocular Surgery	1.41	0.46–4.22	0.53	3.46	1.36–8.72	**<0.01**
Screen time > 6 h/day	2.91	1.89–4.45	**<0.01**	2.74	1.89–4.00	**<0.01**
Oral contraceptive	1.17	0.60–2.28	0.62	1.10	0.60–2.02	0.75
Antidepressant	1.80	1.10–2.96	**0.02**	1.39	0.86–2.24	0.17
Anti-allergy	3.15	1.50–6.58	**<0.01**	1.72	0.80–3.67	0.15
Isotretinoin	0.40	0.03–3.84	0.43	3.04	0.70–12.95	0.13
Diabetes	N/A		-	2.62	0.15–42.83	0.48
Rheumatological disease	2.96	0.23–38.30	0.41	2.03	0.170–24.19	0.56
Cancer treatment	1.15	0.63–2.12	0.65	1.59	0.912–2.77	0.09
Smoking	1.46	0.89–2.38	0.12	1.11	0.68–1.77	0.67
< 6 hours of sleep	1.41	0.46–4.22	0.53	3.46	1.36–8.72	**<0.01**

OR: Odds Ratio, CI: Confidence Interval, OSDI: Ocular Surface Disease Index; WHS: Women’s Health Study; DED: Dry eye disease.

### Clinical evaluation

Participants who met the criteria for DED were invited to proceed to ocular surface tests, and a total of only 54 subjects sought medical evaluation. The results found are displayed in Table 7. This group presented OSDI scores of 38.2 ± 15.5 (95% CI 33.9–42.4). Tear film instability and meibomian gland dysfunction were the most relevant findings. Regarding to TFOS DEWS II diagnostic fluxogram, DED diagnosis is considered in the presence of symptoms plus 1 positive test, such as tear stability or surface staining. Thus, by considering those parameters, DED diagnosis was reached in 64.8% using TBUT results, 5.6% and 1.9% respectively to fluorescein and lisamine staining. No statistical correlations were found among symptoms questionnaires and clinical tests results.

**Table 7:** Results of clinical evaluations of subjects who met the criteria for dry eye disease (DED) as defined by the Dry Eye Syndrome Questionnaire used in the Women’s Health Study (WHS) or the Ocular Surface Disease Index (OSDI).

Variable	Frequency
**Tear break-up time**	
< 10 secs	64.8% (35/54)
>10 secs	35.2% (19/54)
**Expressibility**	
All glands	57.4% (31/54)
3 to 4 glands	31.5% (17/54)
1 to 2 glands	5.3% (1/54)
**Secretion**	
Translucent oil secretion	53.7% (29/54)
Opaque secretion	33.3% (18/54)
Opaque secretion with granules	11.1% (6/54)
Pasty secretion	1.9% (1/54)
**Fluorescein staining (0–15)**	
Up to 4	94.4% (51/54)
5 to 10	5.6% (3/54)
10 to 15	0% (0/54)
**Lisamine green staining (0–9)**	
1 to 3	98.1% (53/54)
4 to 6	1.9% (1/54)
7 to 9	0% (0/54)
**Schirmer’s Test**	
< 10 mm	13% (7/54)
> 10 mm	87% (47/54)

## Discussion

Despite a substantial increase in the number of epidemiological studies evaluating DED worldwide and the extensive focus on this subject, there remains an important lack of information on the prevalence of DED among young people. The current study found the prevalence of DED among a sample of Brazilian undergraduate students to be 34.39% according to the OSDI questionnaire and 23.5% based on the WHS questionnaire. This study also found DED to be associated with a variety of risk factors, a result which underscores the multifactorial etiology of the disease. This study provides the first set of data on DED in a young Brazilian population.

DED among younger patients seems to be progressively more relevant: recent epidemiological studies reinforce clinical impressions that rates of DED are increasing in overall populations in general and among younger adults in particular. Even so, the TFOS DEWS II epidemiology committee most recently found only two population-based studies that included young participants [4]. In the first, a Japanese study evaluated 3,433 high school students between 15 and 18 years of age and found the prevalence of clinically diagnosed DED to be 4.3% in boys and 8.0% in girls; they also found and the prevalence of symptomatic disease to be 21% in boys and 24.4% in girls [6]. In the second study, the prevalence of DED among 1,889 high school students in China was found to be 23.7%, 23.1% of which was based on symptoms, and 1.3% of which was determined by physician diagnosis [9]. Both studies used the WHS questionnaire to evaluate DED.

More recently, additional reports have evaluated DED in undergraduate students. A cross-sectional survey of a group of 813 university students in Mexico determined a DED prevalence of 70.4% as defined by an OSDI score greater than or equal to 12 points; DED was also found to be associated with female sex and smoking. Another study involving 650 undergraduate students in Ghana concluded that the prevalence of symptomatic DED (OSDI≥12) was 44.3%; in this population, the disease was associated with self-medication with over-the-counter eye drops, anti-allergy medication use, oral contraceptive use, windy conditions, areas of low humidity, air-conditioned rooms, and female sex [16]. Among 901 university students from Shanghai, the prevalence of clinically diagnosed DED was estimated to be 10% [5].

The current study defined DED based on a combination of signs and symptoms, with an overall prevalence lower than that of previous studies. Using the same cut-off values for OSDI (≥12), we also detected a higher prevalence of DED (59.64%) relative to the undergraduate students from Mexico and Ghana. Logistic regression analysis found female sex, more than 6 hours per day of screen time, oral contraceptive, antidepressant, and/or anti-allergy medication use, contact lens wear, ocular surgery, and less than 6 hours of sleep per day to be relevant related factors in the Brazilian study population included herein. The clinical evaluation demonstrated mild signs of ocular surface dysfunctions, normal tear volume, tear film instability, and evaporative dry eye.

Population-based surveys around the world have provided interesting numbers. In the United States, overall DED prevalence (all age groups) has been reported to be 6.8%, 2.7% of which represents patients 18 to 34 years of age, and 18.6% of which represents patients older than 75; these findings reflect an increase in prevalence with age [17]. A cross-sectional survey in Ontario, Canada found an overall DED prevalence of 22%. Among those between 18 and 24 years of age, this prevalence was 22.6%, a rate higher than the rate found for patients older than 75 (21.3%) [18]. Both studies reported a significantly higher prevalence in women than in men [17, 18]. A cross-sectional association study that included 79,866 voluntary participants between 20 and 94 years of age in the Netherlands showed that 9.1% of participants reported DED symptoms as measured by the WHS questionnaire. The authors found a relatively high prevalence of symptomatic DED in adults between 20 and 30 years of age and contact lens wear was reported as a noteworthy risk factor for the condition [19].

Our team recently published the first population-based study that evaluated DED symptoms in all five geopolitical regions of Brazil. The WHS questionnaire was applied to more than three thousand participants, and overall DED prevalence was found to be 12.8%. In the current study on younger Brazilian participants, DED prevalence was surprisingly higher: 23.5% according to WHS results. Indeed, the same associated risk factors, such as female sex, prior ocular surgery, and contact lens wear were found to be significant but others must be pointed as relevant factors for younger populations, such as specific medications as isotretinoin, visual display prolonged use and sleeping time [11].

The investigation of risk factors provided herein revealed consistent factors culturally related to young age, such as prolonged visual tasking and more than 6 hours per day of screen time. In a Japanese study on a group of 858 visual display terminal (VDT) users, almost 80% of the subjects exhibited a shorter tear break-up time (TBUT) than blinking interval, as well as a significantly lower Schirmer’s test result [20]. Other studies have mentioned the direct relationship between the use of VDTs and DED in young populations [5, 21]. More than one third of the undergraduate students included in the current study reported sleeping less than 6 hours per day, a routine which produces environmental exposure that is hostile to the ocular surface [22, 23]. Contact lens wear appeared to be a strong risk factor in this young group, as has been reported in other studies [6]. Similarly, in findings consistent with previous studies involving exclusively undergraduate students as well as most previous population-based studies, our results also express a higher prevalence of DED among females. The results obtained herein also show that both a history of ocular surgery and the use of medications such as contraceptives, anti-allergy medications, antidepressants, and isotretinoin derivates represent important risk factors to be investigated further. Post-hoc power calculation was performed to evaluate the statistical power of our sample. Sex and all significant risk factors reached satisfactory power for both tools used, unlike the less prevalent conditions such as diabetes and cancer treatment.

According to the Brazilian Ministry of Education, the country has 6.5 million undergraduate students [24]. The projection of numbers presented in this study might reveal a relevant concern about Brazilian undergraduate students who may suffer from DED. Subjects with higher levels of education are more likely to have diagnosed DED because of their increased access to health care and information on health, as well as of their workplace environments.

Some potential limitations of this study must be pointed out. First, as with any survey, sampling biases must be considered when subjects are voluntarily included. Classes were randomly selected, then, presential visited by the researchers to apply the forms to all present students. Instructions about the study criteria were detailed to avoid the selection bias, as much as possible. Another limitation is the use of self-reporting of symptoms and a previous dry eye diagnosis. Although DED has been recognized as a common ocular problem, its diagnosis remains a challenge due to the lack of gold-standard methods and poor correlations among the most used tests [25]. This study did not consider environmental factors that affect DED, such as air pollutants [19] and desiccating stress [4, 26]; microenvironments are rarely standardized and therefore, represent another limitation herein.

## Conclusions

Our study reveals that dry eye disease is prevalent among young populations; based on the data reported herein. it can be estimated that more than two million Brazilian undergraduate students may suffer from dry eye disease. These data highlight the importance of maintaining awareness of this common condition and continuing research into its causes and treatment.

## Supporting information

S1 Dataset (XLSX)
